# The Simulation of Muscles Forces Increases the Stresses in Lumbar Fixation Implants with Respect to Pure Moment Loading

**DOI:** 10.3389/fbioe.2021.745703

**Published:** 2021-11-22

**Authors:** Matteo Panico, Tito Bassani, Tomaso Maria Tobia Villa, Fabio Galbusera

**Affiliations:** ^1^ Department of Chemistry, Materials and Chemical Engineering “Giulio Natta”, Politecnico di Milano, Milan, Italy; ^2^ IRCCS Istituto Ortopedico Galeazzi, Milan, Italy

**Keywords:** muscles forces, pure moments, spinal fixation, lumbar fixation, realistic loading conditions, simplified loading conditions

## Abstract

Simplified loading conditions such as pure moments are frequently used to compare different instrumentation techniques to treat spine disorders. The purpose of this study was to determine if the use of realistic loading conditions such as muscle forces can alter the stresses in the implants with respect to pure moment loading. A musculoskeletal model and a finite element model sharing the same anatomy were built and validated against *in vitro* data, and coupled in order to drive the finite element model with muscle forces calculated by the musculoskeletal one for a prescribed motion. Intact conditions as well as a L1-L5 posterior fixation with pedicle screws and rods were simulated in flexion-extension and lateral bending. The hardware stresses calculated with the finite element model with instrumentation under simplified and realistic loading conditions were compared. The ROM under simplified loading conditions showed good agreement with *in vitro* data. As expected, the ROMs between the two types of loading conditions showed relatively small differences. Realistic loading conditions increased the stresses in the pedicle screws and in the posterior rods with respect to simplified loading conditions; an increase of hardware stresses up to 40 MPa in extension for the posterior rods and 57 MPa in flexion for the pedicle screws were observed with respect to simplified loading conditions. This conclusion can be critical for the literature since it means that previous models which used pure moments may have underestimated the stresses in the implants in flexion-extension and in lateral bending.

## Introduction

Spinal fixation has become a consolidated treatment for severe degenerative spinal disorders such as adult scoliosis, fixed sagittal imbalance, and high-grade spondylolisthesis (Ha et al., 2008; Casaroli et al., 2019; Galbusera et al., 2020). Despite the generally high success rates of spine surgeries nowadays, biomechanical complications such as hardware failure and loosening are relatively frequent (Kuklo et al., 2001; Tsuchiya et al., 2006; Kebaish, 2010). The literature indeed includes several studies in which different spinal fixation techniques have been investigated in terms of stresses and strains in the instrumentation (Fleischer et al., 2012; Burns et al., 2016; Sutterlin et al., 2016; Casaroli et al., 2019; Cunningham et al., 2019; Zhang and Zhu, 2019), which can be considered as indicators of the risk of biomechanical complications.

Most of the *in vitro* and finite element (FE) studies have been conducted using simplified loading conditions, usually consisting of pure moments, in some cases in combination with compressive forces, which are easier to implement than more realistic conditions involving muscle forces (Stokes and Gardner-Morse, 1995; Wilke et al., 2001; Kim et al., 2014; Zhang and Zhu, 2019). Although several studies confirmed that applying simplified or realistic loading conditions provides the same motion in intact spines (Rohlmann et al., 2009; Han et al., 2011; Zhang and Zhu, 2019), the effect on the instrumentation stresses has never been documented.

As regards the identification of realistic loads, software for musculoskeletal (MSK) modelling such as AnyBody (AnyBody Technology, Aalborg, Denmark) and OpenSim (Stanford University, Stanford, US) provide pre-built models able to predict the muscle forces for any imposed motion of the body segments using algorithms based on inverse dynamics (Bruno et al., 2015; Bassani et al., 2017; Benditz et al., 2018; Liu et al., 2018; Liu et al., 2019). Such models are based on the equations of motion of rigid bodies and cannot therefore be used to estimate the stresses in the implants or in biological structures (Zhu et al., 2013; Arshad et al., 2016; Liu et al., 2018; Bassani et al., 2019). However, the computed muscle forces can be applied as loading condition from the MSK model to the FE model, from which detailed information regarding the hardware stresses can be extracted (Bassani et al., 2017; Liu et al., 2018; Bassani et al., 2019; Liu et al., 2019). Such strategy, coupling FE and MSK models, has never been used to investigate the instrumentation stresses after spinal fixation, and can represent an advantageous approach to determine if the simplified loading conditions consisting of pure moments used in the majority of the available studies are good enough to accurately estimate the hardware stresses in physiological conditions. The aim of this study is therefore to develop and validate coupled FE-MSK models with and without instrumentation and to explore the differences, in terms of hardware stresses, between simplified (pure moment without follower load) and realistic loading conditions.

## Materials and Methods

### Intact Model

**FIGURE 1:**
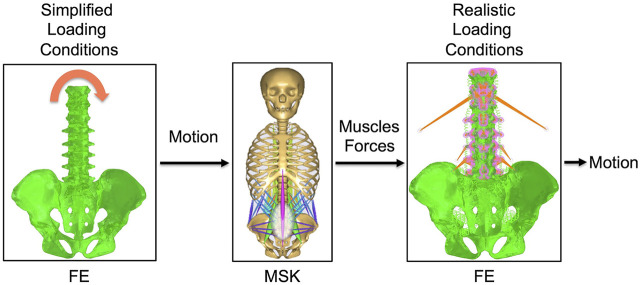
The combination of the intact FE model with the intact MSK model. Simplified loading conditions are applied to the FE model in order to obtain the motion. This motion is imposed as input to the MSK model and muscles forces that contribute to give that motion are predicted by this model. Muscles forces are then applied to the FE model to realistically simulate the loading conditions. The motion under realistic loading conditions is then obtained.

The three-dimensional (3D) geometry of T10-T12 thoracic vertebrae, lumbar vertebrae, and pelvis of the body model from the AnyBody Managed Model Repository (AMMR, version 2.0.0) in the standing posture was used to construct a FE model (Figure 1) made of linear tetrahedral elements after the surfaces were cleaned using MeshLab software (http://www.meshlab.net). Then, the triangular elements of the surfaces of the vertebral endplates were extruded in order to obtain a volume made by tetrahedral elements representing the discs, which were divided into nucleus pulposus and annulus fibrosus based on anatomical data from the literature (Zhong et al., 2014). The annulus included collagen fibers modeled with nonlinear springs; ligaments were also modelled with the same type of elements. The material properties of the bones, intervertebral discs and ligaments were obtained with a calibration procedure based on data reported in the literature (Schmidt et al., 2007). Pure moments of 7.5 Nm in extension, flexion, lateral bending, and axial rotation were applied as simplified loading condition to the upper endplate of T10 through a set of rigid beam elements (Figure 1). The acetabula were completely fixed during all the simulation. The range of motion (ROM) calculated at all levels were then compared to *in vitro* data in order to validate the FE model under simplified loading conditions (Cook et al., 2015; Lindsey et al., 2018).

Then, the obtained ROMs were used as inputs for the MSK model of the thoracolumbar spine with articulated ribcage (Figure 1) developed and validated by Ignasiak et al. (Ignasiak et al., 2016). The muscles simulated in this MSK model were transversus, spinalis, semispinalis, erector spinae, obliquus internus, obliquus externus, psoas major, multifidi, and quadratus lumborum. In this model, the rotational stiffness of the intervertebral joints was calibrated in order to match the linear moment-rotation behavior of the FE model, including the effects of all the joint structures (including facet joint and ligaments). This stiffness does not account for compressive loading or coupling. The pelvis was constrained to the ground. Extension, flexion, and left and right lateral bending movements were simulated by imposing intervertebral rotations matching those calculated with the validated FE model under simplified loading conditions. The muscle forces were calculated for each simulated motion by inverse static analysis (Figure 1).

After that, the obtained muscles forces were modelled at each level (from T10 to the pelvis) in the FE model as concentrated loads to simulate the realistic loading condition (Figure 1), removing the pure moment which implemented the simplified loading conditions; the upper endplate of the T10 vertebra was subjected to a 3D translation equal to that predicted by the MSK model, using a set of rigid beam elements. Moreover, reaction loads from the MSK model were applied to the upper endplate of the T10. Such loading conditions were applied to the FE model for all the investigated motions: extension, flexion, left and right lateral bending. Finally, a validation of the FE model under realistic loading conditions was performed by comparing the ROMs obtained under realistic loading conditions (Figure 1) and the one imposed as input for the MSK model. Ideally, for the validation the ROMs values had to be equal. Moreover, the reaction moment at the upper endplate of T10 was compared between MSK model and FE model in order to provide an extra validation.

### Instrumented Model

From the intact FE model, an instrumented model was derived. This model included a posterior lumbar fixation in which pedicle screws and rods were inserted in the lumbar region between L1 and L5 vertebrae. 3D models of rods and screws were created with commercial software Solidworks (Dassault Systèmes, Waltham, MA, USA). Rods had a circular section with a diameter of 5.5 mm. Pedicle screws had a length of 45 mm and a diameter of 6 mm. The instrumentation was modelled in titanium with an elastic modulus of 110 GPa and a Poisson coefficient of 0.3. Simplified loading conditions and boundary conditions were the same as in the intact model (Figure 1).

Similarly, an instrumented MSK model was created from the intact one. Spinal fusion was modeled by introducing rigid kinematic and kinetic constraints from L1 to L5 vertebrae, guaranteeing rigid connection (no relative motion between vertebrae) and full force and moment transmission (Ignasiak et al., 2018; Ignasiak, 2020). The validation of this model was performed against an *in vivo* study by Rohlmann (Rohlmann et al., 1997), in which bending moments in the posterior rods were measured by means of instrumented implants. Extension, flexion, and left and right lateral bending movements were simulated imposing the intervertebral rotations calculated with the instrumented FE model under simplified loading conditions. From this model, muscle forces were obtained for the four different motions, as for the intact model (Figure 1).

Realistic loading conditions were then simulated in the instrumented model as for the intact model (Figure 1). The FE model under realistic loading conditions was then validated by comparing the intervertebral motion calculated with it with the one imposed as input for the MSK model. As for the intact model, the reaction moment at the upper endplate of T10 was compared between those predicted by the MSK and the FE models in order to provide an extra validation.

### Validation and Output

The outputs of the intact models that were calculated for two types of loading conditions were: 1) the ROM of L1-S1 vertebrae and SIJ; 2) the reaction moment at the upper endplate of T10 joint. The 1) values were used to validate the intact FE model under simplified loading conditions (Cook et al., 2015; Lindsey et al., 2018). 1) and 2) values were used to validate the intact FE model under realistic loading conditions.

The outputs of the instrumented models that were calculated for two types of loading conditions were, in addition to those of the intact model, also: 3) the maximal von Mises stresses in L1-L5 pedicle screws; 4) the maximal von Mises stresses in the posterior rods between the pedicle screws in L1 and L5. The 1) values were used to validate the instrumented FE model under simplified loading conditions. The 1) and 2) values were used to validate the instrumented FE model under realistic loading conditions. The 3) and 4) values were used to compare the hardware stresses between the finite element model under simplified loading conditions and those under realistic loading conditions.

## Results

### Validation of the Intact Model

**FIGURE 2:**
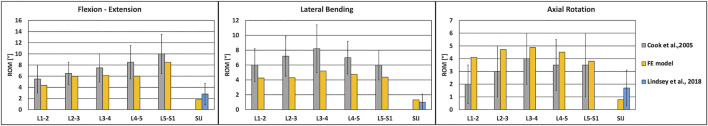
Validation of the intact FE model under simplified loading conditions. Predicted ranges of motion of L1-S1 and sacroiliac joints of the intact model under simplified loading conditions in flexion-extension **(left),** lateral bending (middle) and axial rotation **(right),** as compared with data from *in vitro* experiments, shown as mean and standard deviation (Cook et al., 2015; Lindsey et al., 2018).

The ROMs calculated with the intact FE model under simplified loading conditions showed a tendency toward a higher rigidity with respect to the literature (Cook et al., 2015; Lindsey et al., 2018) (Figure 2); despite this, the predicted values were inside the standard deviations of the *in vitro* data in flexion-extension and axial rotation, except for the L1-L2 ROM in axial rotation which was higher than the corresponding experimental finding. In lateral bending, the ROM was approximately equal to the lower limit of the standard deviations of the *in vitro* studies, except for the SIJ ROM which was in agreement with the value found in the literature.

**FIGURE 3:**
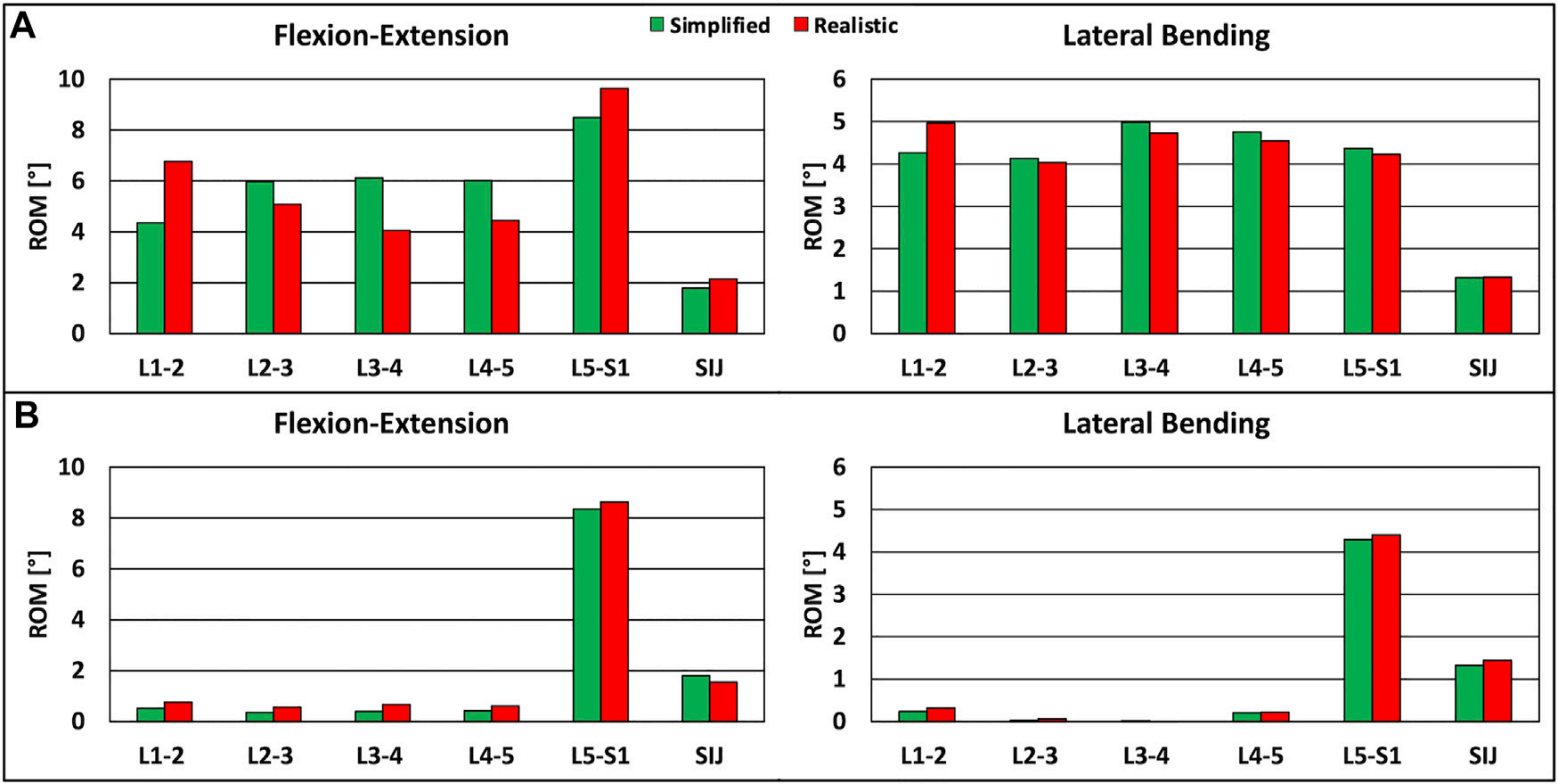
Predicted ranges of motion of L1-S1 and sacroiliac joints under simplified loading conditions and under realistic loading conditions. **(A)** For the intact model in flexion-extension **(left)** and lateral bending **(right).** **(B)** For the instrumented model, in flexion-extension **(left)** and lateral bending **(right)**.

The ROM calculated with the intact model under realistic loading conditions revealed values similar to those calculated with the intact model under simplified loading conditions in flexion-extension and lateral bending, as expected (Figure 3A); nevertheless, some relatively small differences were observed. For instance, negligible differences up to 0.7° were found in lateral bending. The reaction moment at the upper endplate of T10 showed a maximal relative difference of 3.5% between the FE model and the MSK model.

### Validation of the Instrumented Model

The instrumented FE model showed that the ROM of instrumented levels was negligible with respect to the case without instrumentation, as seen in computational and *in vitro* studies (Rohlmann et al., 2007; Dmitriev et al., 2008). The reaction moments at the instrumented levels obtained with the MSK model were inside the range of fixator load measurements assessed *in vivo* (Rohlmann et al., 1997), demonstrating the plausibility of the results.

The comparison of the ROMs under simplified and realistic loading conditions revealed very similar values in the two cases, with small differences up to 0.3° in flexion-extension (Figure 3B). The reaction moment at the upper endplate of T10 showed a negligible difference between the FE model and the MSK model.

### Stresses in the Pedicle Screws

**FIGURE 4:**
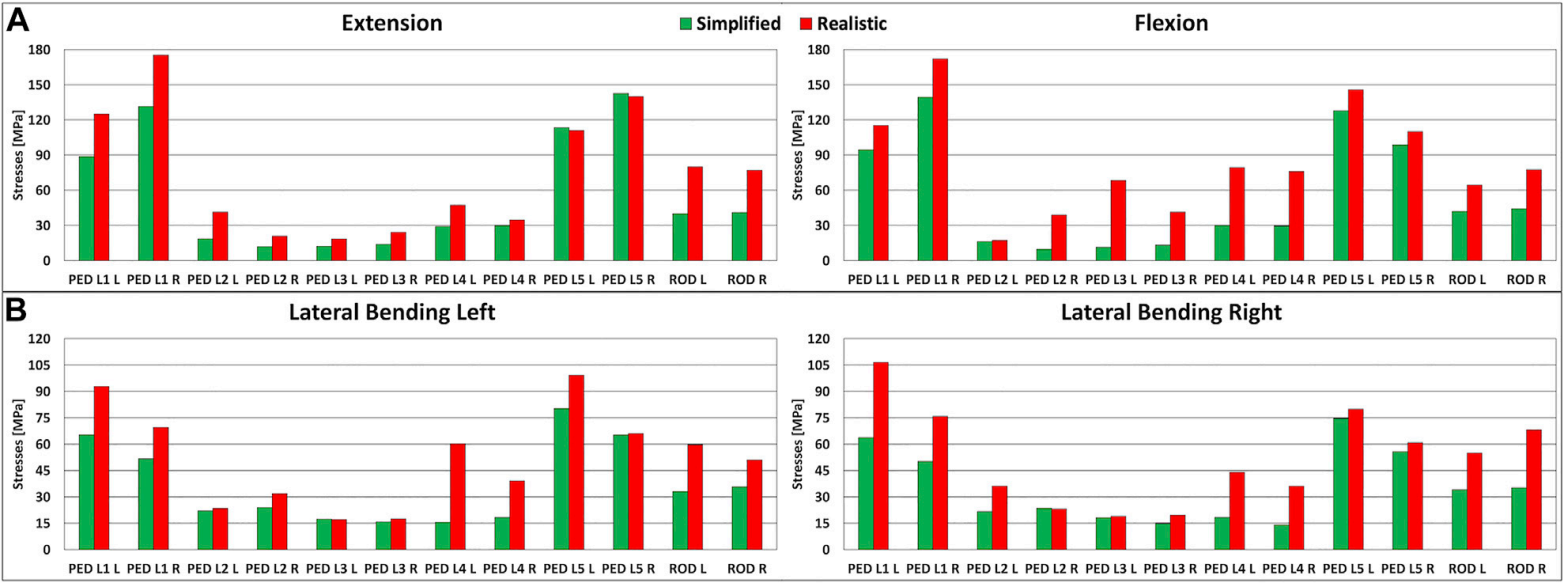
Stresses in the left and right lumbar instrumentation and in the left and right posterior rods. **(A)** Maximal stresses in the L1-L5 pedicle screws and posterior rods in extension **(left)** and in flexion **(right).** **(B)** Maximal stresses in the L1-L5 pedicle screws and posterior rods in lateral bending on the left side **(left)** and on the right side **(right).**

In extension, flexion, lateral bending on the left side, and lateral bending on the right side, the maximum stresses on the L1-L5 pedicle screws were higher when realistic loading conditions were applied to the model (Figures 4A,B). In extension, the maximum stresses for the L1-L4 pedicle screws were higher than the values found with simplified loading conditions. With respect to simplified loading conditions, an increase of 44 MPa (5.6% with respect to the yield stress) for the right pedicle screw in L1 was found under realistic loading conditions. The maximum stresses for the L5 pedicle screws showed more comparable values, with changes up to up to 3 MPa. In flexion, results similar to the extension case were found, but with bigger differences; the highest difference was found for the left pedicle screw in L3 (7.2% with respect to the yield stress) (Figure 4A). For lateral bending, higher stresses on all pedicle screws were predicted when realistic loading conditions were used; the maximal difference resulted to be 45 MPa (5.7% with respect to the yield stress) (Figure 4B).

### Stresses in the Posterior Rods

The maximal stresses on the left and right posterior rods had similar values among simplified and realistic loading conditions (Figures 4A,B). Similar to the pedicle screws, the maximal stresses on the posterior rods were highest when realistic loading conditions were simulated. In flexion-extension, increases exceeding 99% were found with a maximal difference of 40 MPa in extension (5.1% with respect to the yield stress) (Figure 4A). The same trend was observed in lateral bending, but with smaller differences up to 33 MPa (4.2% with respect to the yield stress) (Figure 4B).

## Discussion

This paper presents a preliminary biomechanical comparison between simplified and realistic loading conditions to determine the hardware stresses in a spinal fixation model, aimed at investigating if the implementation of a more realistic loading scenario has the potential to significantly affect the results. Simplified loading conditions consisting of pure bending moments, in some cases combined with compressive loads, are often preferred for *in vitro* and computational testing of spine specimens being easier to implement than more realistic conditions involving muscle forces, while ensuring better reproducibility. Nevertheless, this study demonstrated that applying simplified loads can result in an underestimation of the hardware stresses in the instrumented models, with potential implications about the safety of the implants (Wilke et al., 2001).

In this study, the metrics used to evaluate the importance of the loading conditions were the stresses in the L1-L5 pedicle screws and those in the posterior rods. The results showed that realistic loading conditions increase the stresses in the hardware, up to 57 MPa in flexion (Figures 4A,B). For the pedicle screws in L1-L5, the stresses were higher for all four motions when realistic loading conditions were used. For the posterior rods, higher stresses were found for all conditions with realistic loading conditions (Figures 4A,B). This is a very interesting result because the most common type of biomechanical failure of the fixation is indeed rod breakage (Yamanaka et al., 2015).

Since this approach (coupling FE and MSK modelling) is reported here for the first time for an instrumented spine model, no comparison of the current results with similar existing data can be performed. Despite this, previous studies investigated the validity of simplified loading conditions by comparing *in vivo* measurements with *in vitro* experimental tests. Wilke et al. compared the loads acting on an internal spinal fixator in 10 patients (*in vivo*) with an equivalent *in vitro* simulator under the application of pure bending moments (Wilke et al., 2001). They found good agreement for the loads acting in the internal fixator for axial rotation and lateral bending. For flexion ad extension, reasonable agreement was found only for the healthy spines instrumented with fixators, while for specimens in which a bone graft was implanted in the intervertebral space a lower agreement between *in vivo* and *in vitro* data was found. As regards the proposed FE-MSK approach, other studies in the literature have exploited such combination, but not to investigate instrumented scenarios. For instance, in a computational study by Liu and colleagues a coupled FE-MSK model was used to investigate the load-sharing in the lumbosacral spine, where muscle forces, as predicted by a MSK model, were used as loading conditions for the FE model (Liu et al., 2018; Liu et al., 2019).

This present study has some limitations. Axial rotation was not investigated; however, it is worth considering that the previous papers exploiting coupled FE-MSK models only considered flexion-extension motion, therefore the simulation of lateral bending motion still constitutes an advance with respect to the state-of-the-art (Rohlmann et al., 2006; Liu et al., 2018; Liu et al., 2019). Another limitation was the translation imposed to the most cranial vertebra, which was chosen as boundary condition after verifying that a pure load-controlled simulation driven by muscle forces and the reaction force calculated by the MSK model at the most cranial joint did not lead to convergence. This is however an improvement with respect to the method used by Liu et al., in which the L1 vertebra was subjected to a translation in the direction of the force equal to the one predicted by the MSK model in order to ensure quick convergence of the FE model, but the applied translation was adjusted if the difference of the reaction force in the MSK model and in the FE model was greater than predefined tolerances (Liu et al., 2018; Liu et al., 2019); in the present study, no adjustment was necessary. Besides, only one instrumented configuration was presented in this study while various instrumentation strategies are used in the clinical practice, depending on the condition of the spine of the patient; the simulation of other common configurations is indeed ongoing. Another limitation was that the simplified loading conditions included only pure bending moments without a compressive load mimicking the body weight, such as for example a follower load (Patwardhan et al., 2003). This simplification could justify the difference found in the hardware stresses between the two types of loading conditions, but it should be said that the use of pure moment without compressive loads is very common *in vitro* and computational studies investigating the stresses in the implants (e.g., Wilke et al., 2001; Galbusera et al., 2020), and pure moments have been recommended as the preferred method to test spinal implants in standardized laboratory tests (Wilke et al., 1998). However, we acknowledge that additional studies should be done using a follower load in combination with pure moments in order to simulate another commonly implemented set of simplified loading conditions. Moreover, it should be noted that the motion imposed as input for the MSK model was equal to the validated output of the corresponding FE model under simplified loading conditions, being therefore possibly different from the physiological motion of the spine. In this respect, gait analysis and fluoroscopy can be potential alternatives to determine the motion of the spine to be used as input for the MSK model (Haddas et al., 2018; Breen and Breen, 2020). Finally, it should be noted that the stiffness imposed to the MSK model does not account for compressive loading, but only for the pure moment applied to the FE model.

In conclusion, hardware stresses resulted markedly higher when realistic loading conditions, consisting of muscles forces applied to several vertebrae from T10 to the pelvis, are used instead of simplified loading conditions. This conclusion has relevant biomechanical implications since it means that previous models which used pure moments may have underestimated the stresses in the implants in flexion-extension and in lateral bending. Further studies, using different spinal fixation techniques and follower load, need to be done in order to understand if this combined method is more useful than a simplified one to predict implants failure.

## Data Availability

The raw data supporting the conclusions of this article will be made available by the authors, without undue reservation.

## References

[B1] Arshad R. Zander T. Dreischarf M. Schmidt H. (2016). Influence of Lumbar Spine Rhythms and Intra-abdominal Pressure on Spinal Loads and Trunk Muscle Forces during Upper Body Inclination. Med. Eng. Phys. 38 (4), 333–338. 10.1016/j.medengphy.2016.01.013 26922676

[B2] Bassani T. Stucovitz E. Qian Z. Briguglio M. Galbusera F. (2017). Validation of the AnyBody Full Body Musculoskeletal Model in Computing Lumbar Spine Loads at L4L5 Level. J. Biomech. 58, 89–96. 10.1016/j.jbiomech.2017.04.025 28521951

[B3] Bassani T. Casaroli G. Galbusera F. (2019). Dependence of Lumbar Loads on Spinopelvic Sagittal Alignment: An Evaluation Based on Musculoskeletal Modeling. PloS one 14 (3), e0207997. 10.1371/journal.pone.0207997 30883563 PMC6422292

[B4] Benditz A. Auer S. Spörrer J. F. Wolkerstorfer S. Grifka J. Suess F. (2018). Regarding Loads after Spinal Fusion, Every Level Should Be Seen Separately: a Musculoskeletal Analysis. Eur. Spine J. 27 (8), 1905–1910. 10.1007/s00586-018-5476-5 29352353

[B5] Breen A. Breen A. (2020). Dynamic Interactions between Lumbar Intervertebral Motion Segments during Forward Bending and Return. J. Biomech. 102, 109603. 10.1016/j.jbiomech.2020.109603 31964520

[B6] Bruno A. G. Bouxsein M. L. Anderson D. E. (2015). Development and Validation of a Musculoskeletal Model of the Fully Articulated Thoracolumbar Spine and Rib Cage. J. Biomech. Eng. 137 (8), 081003. 10.1115/1.4030408 25901907 PMC5101035

[B7] Burns C. B. Dua K. Trasolini N. A. Komatsu D. E. Barsi J. M. (2016). Biomechanical Comparison of Spinopelvic Fixation Constructs: Iliac Screw versus S2-Alar-Iliac Screw. Spine Deformity 4 (1), 10–15. 10.1016/j.jspd.2015.07.008 27852493

[B8] Casaroli G. Galbusera F. Chande R. Lindsey D. Mesiwala A. Yerby S. (2019). Evaluation of Iliac Screw, S2 Alar-Iliac Screw and Laterally Placed Triangular Titanium Implants for Sacropelvic Fixation in Combination with Posterior Lumbar Instrumentation: a Finite Element Study. Eur. Spine J. 28 (7), 1724–1732. 10.1007/s00586-019-06006-0 31093749

[B9] Cook D. J. Yeager M. S. Cheng B. C. (2015). Range of Motion of the Intact Lumbar Segment: a Multivariate Study of 42 Lumbar Spines. Int. J. Spine Surg. 9, 5. 10.14444/2005 25785241 PMC4360610

[B10] Cunningham B. W. Sponseller P. D. Murgatroyd A. A. Kikkawa J. Tortolani P. J. (2019). A Comprehensive Biomechanical Analysis of Sacral Alar Iliac Fixation: an *In Vitro* Human Cadaveric Model. J. Neurosurg. Spine 30 (3), 367–375. 10.3171/2018.8.SPINE18328 30611149

[B11] Dmitriev A. E. Gill N. W. Kuklo T. R. Rosner M. K. (2008). Effect of Multilevel Lumbar Disc Arthroplasty on the Operative- and Adjacent-Level Kinematics and Intradiscal Pressures: an *In Vitro* Human Cadaveric Assessment. Spine J. 8 (6), 918–925. 10.1016/j.spinee.2007.10.034 18178528

[B12] Fleischer G. D. Kim Y. J. Ferrara L. A. Freeman A. L. Boachie-Adjei O. (2012). Biomechanical Analysis of Sacral Screw Strain and Range of Motion in Long Posterior Spinal Fixation Constructs. Spine 37 (3), E163–E169. 10.1097/BRS.0b013e31822ce9a7 21857409

[B13] Galbusera F. Casaroli G. Chande R. Lindsey D. Villa T. Yerby S. (2020). Biomechanics of Sacropelvic Fixation: a Comprehensive Finite Element Comparison of Three Techniques. Eur. Spine J. 29 (2), 295–305. 10.1007/s00586-019-06225-5 31773275

[B14] Ha K.-Y. Lee J.-S. Kim K.-W. (2008). Degeneration of Sacroiliac Joint after Instrumented Lumbar or Lumbosacral Fusion: a Prospective Cohort Study Over Five-Year Follow-Up. Spine. Spine 33 (11), 1192–1198. 10.1097/BRS.0b013e318170fd35 18469692

[B15] Haddas R. Ju K. L. Belanger T. Lieberman I. H. (2018). The Use of Gait Analysis in the Assessment of Patients Afflicted with Spinal Disorders. Eur. Spine J. 27 (8), 1712–1723. 10.1007/s00586-018-5569-1 29610989

[B16] Han K.-S. Rohlmann A. Yang S.-J. Kim B. S. Lim T.-H. (2011). Spinal Muscles Can Create Compressive Follower Loads in the Lumbar Spine in a Neutral Standing Posture. Med. Eng. Phys. 33 (4), 472–478. 10.1016/j.medengphy.2010.11.014 21163681

[B17] Ignasiak D. Dendorfer S. Ferguson S. J. (2016). Thoracolumbar Spine Model with Articulated Ribcage for the Prediction of Dynamic Spinal Loading. J. Biomech. 49 (6), 959–966. 10.1016/j.jbiomech.2015.10.010 26684431

[B18] Ignasiak D. Peteler T. Fekete T. F. Haschtmann D. Ferguson S. J. (2018). The Influence of Spinal Fusion Length on Proximal junction Biomechanics: a Parametric Computational Study. Eur. Spine J. 27 (9), 2262–2271. 10.1007/s00586-018-5700-3 30039253

[B19] Ignasiak D. (2020). A Novel Method for Prediction of Postoperative Global Sagittal Alignment Based on Full-Body Musculoskeletal Modeling and Posture Optimization. J. Biomech. 102, 109324. 10.1016/j.jbiomech.2019.109324 31526589

[B20] Kebaish K. M. (2010). Sacropelvic Fixation: Techniques and Complications. Spine 35 (25), 2245–2251. 10.1097/BRS.0b013e3181f5cfae 21102300

[B21] Kim B. S. Lim T.-H. Kwon T. K. Han K.-S. (2014). Feasibility of Compressive Follower Load on Spine in a Simplified Dynamic State: a Simulation Study. Bio-Med. Mater. Eng. 24 (6), 2319–2329. 10.3233/BME-141045 25226932

[B22] Kuklo T. R. Bridwell K. H. Lewis S. J. Baldus C. Blanke K. Iffrig T. M. (2001). Minimum 2-year Analysis of Sacropelvic Fixation and L5-S1 Fusion Using S1 and Iliac Screws. Spine 26 (18), 1976–1983. 10.1097/00007632-200109150-00007 11547195

[B23] Lindsey D. P. Parrish R. Gundanna M. Leasure J. Yerby S. A. Kondrashov D. (2018). Biomechanics of Unilateral and Bilateral Sacroiliac Joint Stabilization: Laboratory Investigation. J. Neurosurg. Spine 28 (3), 326–332. 10.3171/2017.7.SPINE17499 29303472

[B24] Liu T. Khalaf K. Naserkhaki S. El-Rich M. (2018). Load-sharing in the Lumbosacral Spine in Neutral Standing & Flexed Postures - A Combined Finite Element and Inverse Static Study. J. Biomech. 70, 43–50. 10.1016/j.jbiomech.2017.10.033 29153706

[B25] Liu T. Khalaf K. Adeeb S. El-Rich M. (2019). Effects of Lumbo-Pelvic Rhythm on Trunk Muscle Forces and Disc Loads during Forward Flexion: A Combined Musculoskeletal and Finite Element Simulation Study. J. Biomech. 82, 116–123. 10.1016/j.jbiomech.2018.10.009 30389260

[B26] Patwardhan A. G. Havey R. M. Carandang G. Simonds J. Voronov L. I. Ghanayem A. J. (2003). Effect of Compressive Follower Preload on the Flexion-Extension Response of the Human Lumbar Spine. J. Orthop. Res. 21 (3), 540–546. 10.1016/S0736-0266(02)00202-4 12706029

[B27] Rohlmann A. Bergmann G. Graichen F. Weber U. (1997). Comparison of Loads on Internal Spinal Fixation Devices Measured *In Vitro* and *In Vivo* . Med. Eng. Phys. 19 (6), 539–546. 10.1016/s1350-4533(97)00018-0 9394902

[B28] Rohlmann A. Bauer L. Zander T. Bergmann G. Wilke H.-J. (2006). Determination of Trunk Muscle Forces for Flexion and Extension by Using a Validated Finite Element Model of the Lumbar Spine and Measured *In Vivo* Data. J. Biomech. 39 (6), 981–989. 10.1016/j.jbiomech.2005.02.019 16549091

[B29] Rohlmann A. Burra N. K. Zander T. Bergmann G. (2007). Comparison of the Effects of Bilateral Posterior Dynamic and Rigid Fixation Devices on the Loads in the Lumbar Spine: a Finite Element Analysis. Eur. Spine J. 16 (8), 1223–1231. 10.1007/s00586-006-0292-8 17206401 PMC2200767

[B30] Rohlmann A. Zander T. Rao M. Bergmann G. (2009). Applying a Follower Load Delivers Realistic Results for Simulating Standing. J. Biomech. 42 (10), 1520–1526. 10.1016/j.jbiomech.2009.03.048 19433325

[B31] Schmidt H. Heuer F. Drumm J. Klezl Z. Claes L. Wilke H.-J. (2007). Application of a Calibration Method Provides More Realistic Results for a Finite Element Model of a Lumbar Spinal Segment. Clin. Biomech. 22 (4), 377–384. 10.1016/j.clinbiomech.2006.11.008 17204355

[B32] Stokes I. A. F. Gardner-Morse M. (1995). Lumbar Spine Maximum Efforts and Muscle Recruitment Patterns Predicted by a Model with Multijoint Muscles and Joints with Stiffness. J. Biomech. 28 (2), 173–186. 10.1016/0021-9290(94)e0040-a 7896860

[B33] Sutterlin C. E. 3rd Field A. Ferrara L. A. Freeman A. L. Phan K. (2016). Range of Motion, Sacral Screw and Rod Strain in Long Posterior Spinal Constructs: a Biomechanical Comparison between S2 Alar Iliac Screws with Traditional Fixation Strategies. J. Spine Surg. 2 (4), 266–276. 10.21037/jss.2016.11.01 28097243 PMC5233850

[B34] Tsuchiya K. Bridwell K. H. Kuklo T. R. Lenke L. G. Baldus C. (2006). Minimum 5-year Analysis of L5-S1 Fusion Using Sacropelvic Fixation (Bilateral S1 and Iliac Screws) for Spinal Deformity. Spine 31 (3), 303–308. 10.1097/01.brs.0000197193.81296.f1 16449903

[B35] Wilke H.-J. Wenger K. Claes L. (1998). Testing Criteria for Spinal Implants: Recommendations for the Standardization of *In Vitro* Stability Testing of Spinal Implants. Eur. Spine J. 7 (2), 148–154. 10.1007/s005860050045 9629939 PMC3611233

[B36] Wilke H.-J. Rohlmann A. Neller S. Schulthei M. Bergmann G. Graichen F. (2001). Is it Possible to Simulate Physiologic Loading Conditions by Applying Pure Moments? Spine 26 (6), 636–642. 10.1097/00007632-200103150-00014 11246374

[B37] Yamanaka K. Mori M. Yamazaki K. Kumagai R. Doita M. Chiba A. (2015). Analysis of the Fracture Mechanism of Ti-6Al-4V Alloy Rods that Failed Clinically after Spinal Instrumentation Surgery. Spine 40 (13), E767–E773. 10.1097/BRS.0000000000000881 25785960

[B38] Zhang H. Zhu W. (2019). The Path to Deliver the Most Realistic Follower Load for a Lumbar Spine in Standing Posture: A Finite Element Study. J. Biomech. Eng. 141 (3), 031010. Advance online publication. 10.1115/1.4042438 30615030

[B39] Zhong W. Driscoll S. J. Wu M. Wang S. Liu Z. Cha T. D. (2014). *In Vivo* morphological Features of Human Lumbar Discs. Medicine 93 (28), e333. 10.1097/MD.0000000000000333 25526494 PMC4603132

[B40] Zhu R. Zander T. Dreischarf M. Duda G. N. Rohlmann A. Schmidt H. (2013). Considerations when Loading Spinal Finite Element Models with Predicted Muscle Forces from Inverse Static Analyses. J. Biomech. 46 (7), 1376–1378. 10.1016/j.jbiomech.2013.03.003 23540724

